# Cross-Cultural Evidence for Apparent Racial Outgroup Advantage: Congruence between Perceived Facial Aggressiveness and Fighting Success

**DOI:** 10.1038/s41598-018-27751-0

**Published:** 2018-06-27

**Authors:** Vít Třebický, S. Adil Saribay, Karel Kleisner, Robert Mbe Akoko, Tomáš Kočnar, Jaroslava Varella Valentova, Marco Antonio Correa Varella, Jan Havlíček

**Affiliations:** 1 Faculty of Science, Charles University, Prague, Czech Republic; 2 National Institute of Mental Health, Klecany, Czech Republic; 3 Faculty of Arts and Sciences, Boğaziçi University, Istanbul, Turkey; 4 Faculty of Social and Management Sciences, University of Buea, Buea, Cameroon; 5 Institute of Psychology, University of São Paulo, São Paulo, Brazil

## Abstract

Research into face processing consistently shows an outgroup disadvantage in areas such as recognition memory and emotional identification. Potential ingroup advantage with respect to inferences regarding personality and behavioural outcomes, on the other hand, has not yet been studied. In the present study, we used the faces of male professional mixed martial arts (MMA) fighters of apparent African, European, or mixed-race origin as targets and males from four distant populations that vary in ethnic composition as perceivers. We compared the perceivers’ inferences about targets’ aggressiveness with the fighters’ actual performance in professional MMA championships. Surprisingly, across three distant populations used in the study (Cameroon, Czech Republic, and Turkey), perceivers’ inferences based on face rating were more congruent with real-world performance for targets belonging to an apparent racial outgroup (as opposed to ingroup). In an ethnically mixed population (Brazil), perceivers showed the lowest congruence for apparently mixed-race targets. It thus seems that the outgroup disadvantage observed in other face processing domains does not carry over to inferences about aggressive behavioural outcomes. In fact, it seems that this relationship is, if anything, reversed.

## Introduction

Each human face presents a unique constellation of morphological features. Based on a face, perceivers make inferences about the individual identity, social categories (age, gender, ethnic origin), affective expressions, and personality characteristics of its owner^1^. Interestingly, it turns out that perceivers’ inferences regarding behavioural tendencies and personality traits are to some degree congruent with the face owner’s self-reports^2^ and certain objective standards^3^.

Face processing is, however, also affected by various biases. The most commonly described bias is the tendency to recognise faces belonging to people from one’s apparent racial ingroup better than faces of persons from other groups^4^, referred to as the cross-race effect (CRE). This effect may be in part due to expertise, as indicated by the fact that increasing familiarity and contact with outgroups leads to a decrease in the CRE^5,6^, and even categorical inferences about targets (e.g., their sexual orientation) become more accurate^7^. Efficiency with which minimal^8^ and cultural ingroup faces are processed^9,10^ may also be due to greater motivation to attend to ingroup faces. Various accounts of the CRE emphasise these factors (such as expertise and motivation) and commonly assume that they lead to more efficient and careful encoding of ingroup faces, which, in turn, results in the CRE^11^.

Research into other aspects of face processing also consistently indicates an outgroup disadvantage. While outgroup faces may be categorised more rapidly as such^12^, this effect is closely linked to task features and may be part of a ‘quick and dirty’ approach that helps to disengage attention from outgroup faces as soon as possible^13^. Indeed, unless there is a specific reason to attend to outgroups, perceivers tend to preferentially direct their *attention* toward ingroup members’ faces^14^ (see Discussion). This attention bias affects especially the eye area, the most social-cognitively significant part of human face^15^, and it seems to be one of the reasons underlying the CRE^14^.

Likewise, *inference of emotions from facial expressions* is also more accurate for minimal and cultural ingroup, as opposed to outgroup, targets^16,17^. In the sole exception to this well-established pattern, Young^18^ quite unexpectedly found that perceivers were both more accurate and faster in distinguishing fake from genuine smiles in minimal outgroup targets. Hwang and Matsumoto^19^ have observed that American perceivers of European origin were more accurate in detecting deceitful communication of Chinese (as opposed to American of European origin) targets from interview videos. The design of this study, however, involved other factors and the pattern was observed only under specific conditions and not as an overall effect.

Finally, in research examining *categorisation accuracy*, where differences are present, perceivers tend to favour the ingroup. For instance, both Czechs and U.S. Americans are more accurate in differentiating homosexuals, based on thin slices of behaviour, among their respective co-nationals^20^. Judgments on sexual orientation based on facial images did not, however, reveal either ingroup or outgroup advantage^21^.

The results of existing research on accuracy of facial inferences about *personality or behavioural outcomes* which examined the effects of targets’ and perceivers’ group membership form a rather inconsistent pattern. Some studies focused on impressions (but not accuracy) found that perceivers from various cultures (e.g., U.S. Americans and Koreans) converge on facial impressions (such as dominance or attractiveness) owing partly to commonly held appearance-related stereotypes^22^. Even with such stereotypes in place, however, within-culture agreement on judgments regarding faces from racial outgroups is low, which indicates difficulty of processing other-race faces^23^. Some findings hint at a greater inferential accuracy for ingroup targets^24^, others for outgroup^25^ targets. Nonetheless, a particularly relevant study by Short *et al*.^26^ found that Chinese and Caucasian child and adult perceivers did not differ in their tendency to rely on facial features in judging ethnic ingroup and outgroup targets’ propensity for aggression, even though accuracy was not assessed.

## The Present Study

Research showing an ingroup advantage/outgroup disadvantage in face processing (recognition, attention, categorisation, and identification of emotions) strongly suggests that congruence between *facial inferences* and *behavioural outcome*s should, if anything, be higher for apparent racial ingroup than outgroup faces. The present study tested this hypothesis with male targets and perceivers in four disparate cultures. As targets, we used male professional Mixed Martial Arts (MMA) fighters of different apparent races (European, African, and Mixed), because this allowed us to use both standardised facial images and an objective standard for congruence between inference and real-world outcome, namely actual fighting performance. We predicted a greater congruence between these inferences and fighting performance when African (Cameroonian) raters rated African-looking faces and Europeans (Czechs and Turks) rated European-looking faces. Given the ethnic diversity and prevalence of people of mixed-race (European and African) descent in Brazil, neither African- nor European-looking faces clearly represent ingroup or outgroup in that culture. We have therefore expected that among Brazilian perceivers, there would be no differences in accuracy with respect to perceived race.

## Methods

The data collection procedure was approved by the Institutional Review Board of the Charles University, Faculty of Science (Ref. num. 2013/14). All methods were performed in accordance with the relevant guidelines and regulations.

### Material

Stimuli set consisted of 54 male facial photographs selected from available portraits of Ultimate Fighting Championship (UFC) MMA fighters (images freely accessible on the official website of the UFC; www.ufc.com; downloaded in July 2012). Portraits were selected based on criteria, such as facing directly into the camera, absence of beard or moustache, and a history of at least 2 fights in the UFC (as per Třebický *et al*.^3^). Images were then sorted by two independent raters into 3 categories based on apparent race, which resulted in 18 images of men of apparent African origin, 18 images of men of apparent European origin, and 18 images of apparent mixed-race (Mixed) origin. Each category contained the same number of fighters within a weight class (9 in bantamweight, 6 in featherweight, 12 in lightweight, 6 in welterweight, 6 in middleweight, 9 in light heavyweight, and 6 in heavyweight). For each fighter, we obtained data about the number of fights he participated in (Mean = 8.6, SD = 6.83, Range = 2–27) and fights he won (Mean = 5.68, SD = 5.19, Range = 0–22) in the UFC. The images were subsequently standardised with respect to the position of the face within the frame (e.g. same position on a vertical axis), and the background colour was set to grey (RGB 128, 128, 128). To account for differences in the number of fights between fighters, we computed fighting success as a proportion of wins relative to the total number of fights (Mean = 0.59, SD = 0.25). We did not include female fighters in our study because of a low number of fighters and insufficient data regarding the number of fights at the time of data collection.

### Participants

In total, 136 male students (mean age = 25.82ys, SD = 4.78; range = 18–49) from the University of São Paulo (São Paulo, Brazil) were invited to participate in the research online. Ninety participants from two different sites in Cameroon: 47 men (mean age = 23.43ys; SD = 5.81; range 18–41) from a rural community of Big Babanki (located in the Northwest Region) were recruited by a local research assistant (Ernest Vunan and JH), while 43 male students (mean age = 22.07ys; SD = 2.14; range 18–30) from the University of Buea, Buea, were recruited with the help of a local researcher (RMA and KK, TK, VT). Both Cameroonian samples participated in our research offline, on site. The two data collection sites did not differ in their ratings, which is why we merged them into one Cameroonian sample (N = 90) (for details, see Supplementary materials). The sample from the Czech Republic (N = 80) consisted of two data collections carried out during 2015 and 2017. An earlier Czech sample collected in 2013 showed effects incongruent with other cultures (see Table S4 in Supplementary materials). We found this suspicious and collected two other Czech samples (reported below), which both behaved differently from the initial Czech sample. This convinced us to treat the earlier as an exception. The 2015 sample consisted of 46 male students (mean age = 24.1ys; SD = 3.86; range 18–33) from the Charles University, Prague. The 2017 sample consisted of 34 male students (mean age = 25.88ys; SD = 7.68; range 18–49), also from the Charles University, Prague. Czech participants were invited to take part in the research online. The two data collection samples did not differ in their ratings, which is why we merged them into one Czech sample (N = 80) (for details, see Supplementary materials). In Turkey, 143 male students (mean age = 20.9ys; SD = 1.84; range 18–29) from Boğaziçi University, Istanbul were invited to participate in the research online. All participants provided their informed consent by clicking on the ‘I agree’ button to consent with their participation in the study.

### Procedure

In Brazil, Czech Republic, and Turkey, rating was carried out using an online survey platform (Qualtrics, Provo, UT), while in Cameroon, where access to internet was limited, we used an offline rating software. Participants completed a short demographic questionnaire. Then they were presented with the whole set of images in a random order and asked to rate the aggressiveness of each face^3^ using a 7-point verbally-anchored scale (ranging from 1 – not aggressive at all, to 7 – very aggressive). A mean perceived aggressiveness rating was calculated separately for each category of stimuli (African, European, and Mixed), resulting in 3 scores for each participant. These scores were subsequently converted into z-scores to account for differences in scale use between participants within each population. We used aggressiveness rating as a perceptual proxy for inference regarding fighting success because our previous study had shown that perceived aggressiveness does predict actual fighting success in UFC and closely correlates with perceived fighting ability^3^.

### Statistical analyses

All analyses were performed using IBM SPSS Statistics for Windows, version 23 (IBM Corp., Armonk, N.Y., USA). Effect sizes for two-way mixed ANOVA are reported in partial η^2^. Aggressiveness ratings of all fighters were correlated with their fighting performance, and the resulting correlation coefficients were used as *congruence scores* in the corresponding analyses.

Figures were generated in BoxPlotR and post-produced in Adobe Photoshop CC 2018.

### Data availability

Datasets generated and analysed during the current study are available in the Open Science Framework, https://osf.io/wec83/.

## Results

Results indicating inter-rater reliability of aggressiveness rating based on raw scores from each country are shown in Table 1. In the Brazilian sample, UFC and the MMA in general are widely popular and fighters are well-known persons. For Brazilian participants, rating procedure therefore included an option where raters could indicate recognition for each face. Where a rater indicated recognition, his rating for that face was skipped. For Cronbach’s Alpha analyses, the resulting missing values were thus substituted by the mean ratings from the Brazilian sample for the image in question.

**Table 1:** Inter-rater reliability (Cronbach’s alpha) of aggressiveness ratings.

Country	Stimuli		
	African	European	Mixed
Brazil (*N* = 136)	0.986	0.987	0.989
Cameroon, Babanki (*N* = 47)	0.870	0.809	0.762
Cameroon, Buea (*N* = 43)	0.955	0.944	0.934
Czech Republic, 2015 (*N* = 46)	0.970	0.965	0.963
Czech Republic, 2017 (*N* = 34)	0.928	0.921	0.947
Turkey (*N* = 143)	0.989	0.981	0.988

Two-way mixed ANOVA with congruence scores as a dependent variable and stimuli category (within-subjects) and rater’s country (between-subjects) as independent variables was carried out (the same analysis was conducted using aggressiveness assessments as a dependent variable, see Supplementary materials).

**Figure 1:**
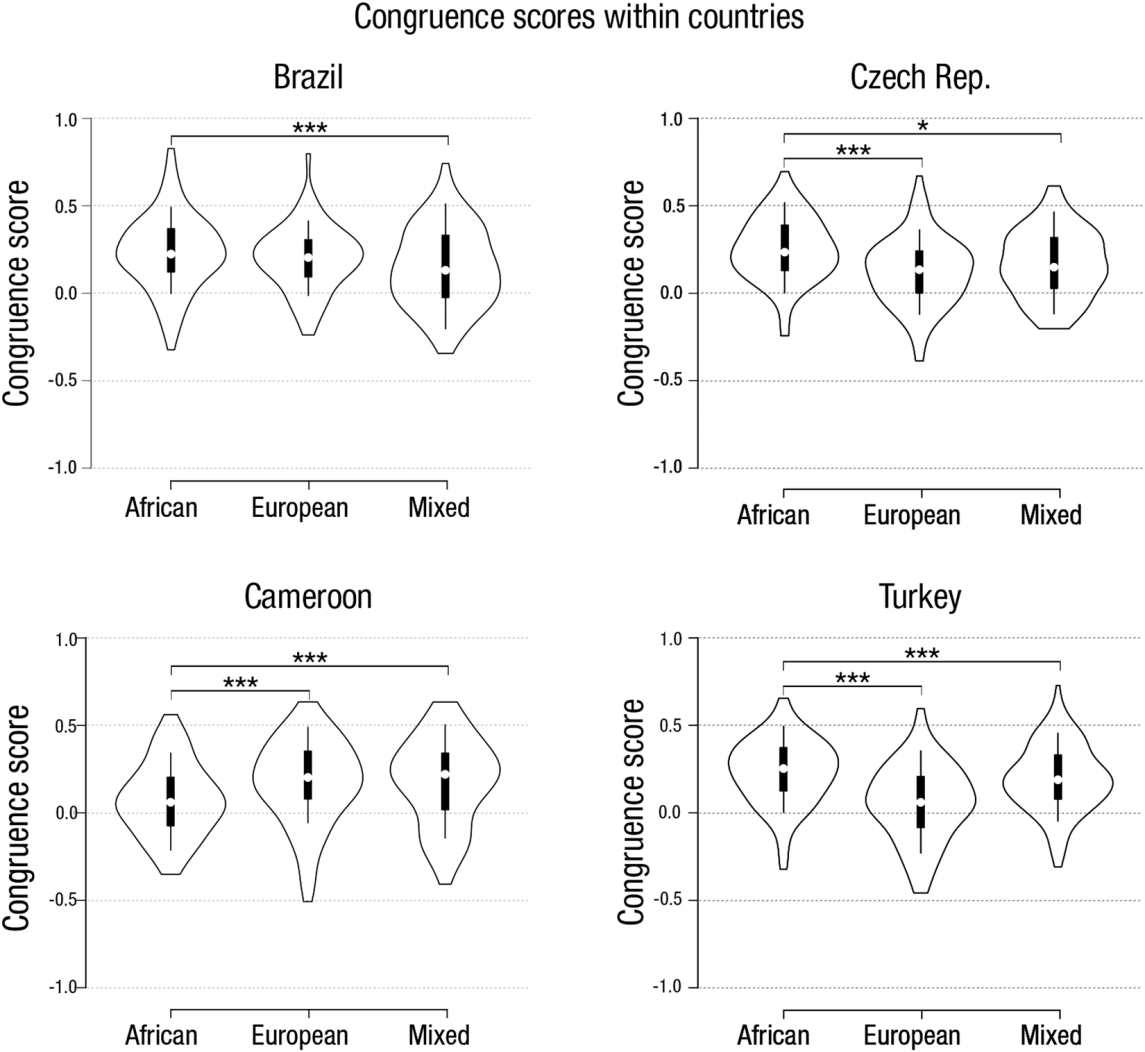
Violin and boxplots for differences in congruence scores. Note: Violin plots represent congruence scores. Box limits indicate the 25^th^ and 75^th^ percentiles, whiskers extend 1.5 times the interquartile range from the 25^th^ and 75^th^ percentiles. Asterisks stand for significance levels; *** for p < 0.001, ** for p < 0.01, * for p < 0.05.

Results, including *post-hoc* tests, are summarised in Tables 2 and 3. The analysis showed a significant main effect of the stimuli category (F_1.965,870.591_ = 8.886, *p* < 0.001, η_*p*_^2^ = 0.02) on congruence scores. The category of apparent African origin was rated with significantly higher congruence scores (i.e., agreement between perceived aggressiveness and actual fighting success), than either the apparently European or the apparently Mixed origins, which did not significantly differ from one another (Table 2). This main effect was qualified by a statistically significant interaction between stimuli category and rater’s country on congruence scores (F_5.896,870.591_ = 12.462, *p* < 0.001, η_*p*_^2^ = 0.078) (Fig. 1). To understand the nature of the interaction, we ran pairwise comparisons with Bonferroni correction. In the Brazilian sample, congruence scores were higher for African than for Mixed faces (*p* < 0.001) and there were no other significant differences. In Cameroon, apparently African faces received significantly lower congruence scores than either apparently European or Mixed faces (*p*s < 0.002), which did not differ from each other. In the Czech Republic, apparently European and Mixed faces received significantly lower congruence scores than apparently African faces (*p* < 0.001 and p = 0.022, respectively), but they did not differ from each other. In Turkey, apparently European faces received significantly lower congruence scores than apparently African or Mixed faces (*p*s < 0.001), but the latter did not differ from one another (Table 3).

**Table 2:** Differences in congruence scores - Stimulus category descriptive statistics and Post Hoc comparison.

	Stimuli	Congruence Score		95% Confidence intervals		
		Mean	SE	Lower limit	Upper limit	
Stimulus category descriptives	African	0.197	0.010	0.178	0.216	
	European	0.136	0.010	0.116	0.156	
	Mixed	0.169	0.010	0.148	0.189	
	**Stimuli**	**Bonferroni Post Hoc comparison**				***p***
		**Mean difference**	**SE**	**95% Confidence intervals**		
				**Lower limit**	**Upper limit**	
Pairwise comparisons	African - European	0.060	0.014	0.028	0.093	<0.001
	African - Mixed	0.028	0.014	−0.006	0.062	0.145
	European - Mixed	−0.032	0.015	−0.069	0.004	0.102

**Table 3:** Differences in congruence scores - Country × Stimulus category descriptive statistics and Post Hoc comparison.

	Congruence Score						
	Country	Stimuli	Mean	SE	95% Confidence intervals		
					Lower limit	Upper limit	
Country × stimulus category descriptives	Brazil	African	0.231	0.017	0.198	0.265	
		European	0.196	0.018	0.161	0.232	
		Mixed	0.143	0.018	0.107	0.179	
	Cameroon	African	0.066	0.021	0.025	0.107	
		European	0.166	0.022	0.123	0.209	
		Mixed	0.170	0.022	0.126	0.214	
	Czech Rep.	African	0.252	0.022	0.208	0.295	
		European	0.124	0.023	0.078	0.170	
		Mixed	0.164	0.024	0.117	0.211	
	Turkey	African	0.238	0.017	0.205	0.270	
		European	0.059	0.018	0.024	0.093	
		Mixed	0.198	0.018	0.162	0.233	
	**Bonferroni Post Hoc comparison**						
	**Country**	**Stimuli**	**Mean difference**	**SE**	**95% Confidence intervals**		***p***
					**Lower limit**	**Upper limit**	
Pairwise comparisons	Brazil	African - European	0.035	0.024	−0.023	0.093	0.432
		African - Mixed	0.089	0.025	0.028	0.149	0.001
		European - Mixed	0.053	0.027	−0.011	0.118	0.143
	Cameroon	African - European	−0.100	0.029	−0.171	−0.029	0.002
		African - Mixed	−0.104	0.031	−0.177	−0.030	0.002
		European - Mixed	−0.004	0.033	−0.083	0.075	1.000
	Czech Rep.	African - European	0.128	0.031	0.053	0.203	<0.001
		African - Mixed	0.087	0.033	0.009	0.165	0.022
		European - Mixed	−0.041	0.035	−0.125	0.043	0.739
	Turkey	African - European	0.179	0.023	0.122	0.235	<0.001
		African - Mixed	0.040	0.024	−0.019	0.099	0.303
		European - Mixed	−0.139	0.026	−0.202	−0.076	<0.001

## Discussion

Contrary to our hypothesis, congruence between facial inferences and real-world performance in three distant cultures (Cameroon, Czech Republic, and Turkey) indicates an outgroup advantage. In an ethnically heterogenous and mixed culture (Brazil), congruence was the lowest for the apparently mixed-race targets. To the best of our knowledge, only three sets of earlier studies have examined the accuracy of face-based inferences regarding personality or behavioural outcome in perceivers from different cultures and both ingroup and outgroup targets^24,25,27^. Interestingly, Na *et al*.^25^ have observed a weak outgroup advantage in accuracy. In their study, competence judgments of American politicians’ faces by Korean (vs. American) perceivers and competence judgments of Korean politicians’ faces by American (vs. Korean) perceivers were stronger predictors for respective election results. Young^18^ had also observed an outgroup advantage: he performed two experiments focused on identifying fake smiles using minimal groups. It thus seems that at least in some areas related to competition and cooperation, there may be an outgroup advantage in inferential accuracy.

Our results contrast with research that demonstrated an ingroup advantage in other areas of face processing. Decades of research on social cognition^28^ have established that ingroup members are more likely to be individuated (i.e., perceivers are especially attentive to facial features which distinguish ingroup faces from one another), whereas outgroup members are more likely to be processed categorically (perceivers are attentive to facial features common across outgroup faces). As a consequence, people tend to have more detailed impressions of apparent racial ingroup (as opposed to outgroup) faces^29^. Such individuation should logically result in a more accurate prediction of a target’s dispositions than categorisation.

Did our perceivers individuate the outgroup targets more than the ingroup ones? It has been shown that perceivers tend to be motivated to paying special attention to outgroup faces that may present a potential threat^30,31^. This effect is further amplified by activation of a self-protection goal^32^ and may well be exclusive to male faces^33^. Given that in our study, perceivers were instructed to focus on *aggressiveness in male faces*, it is thus possible that they were motivated to process outgroup faces more carefully (i.e., to focus on individuation) in order to determine each target’s threat potential. This would amount to a self-protective response. Our results may therefore represent an extension of motivated face processing directed at outgroup targets, investigated in earlier face memory research^4^, to the domain of inferential accuracy. Asking perceivers to judge aggressiveness may have primed a certain level of threat and guardedness (especially since the targets were male fighters), just as Young’s^18^ experiments where perceivers were asked to detect fake smiles may have activated mistrust and guardedness (especially toward outgroup targets). Prima facie, one could expect that such priming might lead to an indiscriminately heightened attribution of aggressiveness to an outgroup. Interestingly, our results show no differences in perceived aggressiveness level attributed to any particular group of faces. It is yet to be seen, however, whether our findings could be generalised to contexts other than aggression or physical competition and to female faces and female raters. This remains a task for future research.

Another way to interpret our findings is to frame them as an ingroup disadvantage and to investigate why perceivers may find it more difficult to process ingroup faces. The greater propensity to individuating ingroup faces may have somewhat paradoxically detracted from the accuracy of inferences by introducing additional cognitive processing^29^, which diluted the immediate impact of valid facial cues on perception. Similar detrimental effects on deliberation have been reported in other domains such as decision making^34^.

As mentioned above, the initial Czech sample collected in 2013 showed effects inconsistent with other cultures. We have therefore collected two other Czech samples which show patterns consistent with the other datasets. We have as yet no rational explanation for why the initial Czech sample was difficult to reconcile with the rest of the data. One might speculate whether it could have been related to the media attention given to the so-called ‘immigration crisis’ which escalated in Europe in 2015. Nevertheless, robustness of our current findings should be reassessed in the future studies.

An important limitation of the current study was its failure to assess inter-ethnic contact among the respondents. Based on societal demographics, we assumed that Czechs and Turks have relatively little exposure to faces of African appearance, while Cameroonians are the opposite, and Brazilians have approximately equal exposure to faces of European and African appearance. It has, however, been established that attenuation of the CRE can be the result of inter-ethnic contact measured at an individual level^35^. Future research should thus examine whether contact plays a similar role for accuracy of facial inferences with respect to behavioural outcomes. If accuracy decreases with increased contact (as the cross-cultural differences seem to suggest), this would present an interesting explanatory challenge.

Data regarding the ethnic origin of the individual fighters were not available on UFC website. Moreover, we gathered no data on how raters perceive the depicted faces in terms of their ethnic similarity. Nevertheless, given a generally high contrast between faces of European and African appearance, we believe that the assumption about perceived ethnic similarity is justified.

Because social-motivational factors can significantly affect face processing^17^, it is possible that perceivers’ own ethnic identity (and their categorisation of target faces into ethnic ingroup and outgroup with subsequent biases triggered by such a categorisation) may have influenced the current findings^9^. Given that the extent to which perceivers identify with their ingroup is known to moderate face processing^14,36^, future research should test the influence of perceivers’ ethnic identity on the accuracy of inferences made from ingroup and outgroup faces.

Finally, facial morphology differs between groups of people, such as heterosexuals and homosexuals^37,38^, and is related to behaviour^39^. Future research should thus investigate the facial cues our perceivers used to judge aggressiveness and determine which of these cues actually increased the accuracy of perceivers’ inferences. To wit, it is possible that biased processing of faces led to increased accuracy merely accidentally. It is known that, for instance, perceivers focus more attention on the eye area of ingroup target faces than outgroup faces^15^. If our perceivers were more likely to attend to other areas of outgroup faces than the eye region, and if those areas happened to contain cues that aided accurate inferences (e.g., relative facial width), their accuracy for outgroup faces may have been higher *not despite but because of* biased outgroup face processing.

Within the still little body of research that examined face processing across different groups of both targets and perceivers and applied some real-world accuracy criterion, reports of an outgroup advantage are beginning to emerge^18,25^, countering evidence for an outgroup disadvantage in other face-processing domains. Our findings suggest that these recent reports of an outgroup advantage have captured a real and replicable phenomenon that may have as yet escaped the researchers’ attention. Future research should try to uncover the mechanisms and moderators of this outgroup advantage in inferential accuracy.

## Electronic supplementary material

Supplementary materials
