# Supplementary material for: Cross-Cultural Evidence for Apparent Racial Outgroup Advantage: Congruence between Perceived Facial Aggressiveness and Fighting Success
Source: Sci Rep. 2018 Jun 27;8:9767. doi: 10.1038/s41598-018-27751-0 (PMC6021408; doi:10.1038/s41598-018-27751-0)
Supplement: Supplementary file 1 — Supplementary materials [file 41598_2018_27751_MOESM1_ESM.docx]

Supplementary materials: Cross-Cultural Evidence for Apparent Racial Outgroup Advantage: Congruence between Perceived Facial Aggressiveness and Fighting Success

Vít Třebický ^a, b, ‡ *^

S. Adil Saribay ^c, ‡^

Karel Kleisner ^a, b^

Robert Mbe Akoko ^d^

Tomáš Kočnar ^a^

Jaroslava Varella Valentova ^e^

Marco Antonio Correa Varella ^e^

Jan Havlíček ^a, b^

^a^ Faculty of Science, Charles University, Czech Republic

^b^ National Institute of Mental Health, Czech Republic

^c^ Faculty of Arts and Sciences, Boğaziçi University, Istanbul, Turkey

^d^ Faculty of Social and Management Sciences, University of Buea, Cameroon

^e^ Institute of Psychology, University of São Paulo, Brazil

‡ The first two authors contributed equally to this work.

* Address correspondence to Vít Třebický, Faculty of Science, Charles University, Viničná 7, Prague 2, 128 44, Czech Republic; [vit.trebicky@natur.cuni.cz](mailto:vit.trebicky@natur.cuni.cz)

**Supplementary materials**

**Differences in ratings within the Cameroonian and the Czech samples**

To assess whether the two collection sites from Cameroon and the two data collection periods in the Czech Republic differ from one another, two-tailed independent sample t-tests were carried out. Results showed no significant differences between the Babanki and Buea samples (from Cameroon) in aggressiveness rating or congruence score. The same was found for the two data collection periods in the Czech Republic. For further analyses, we have therefore merged the ratings into one Cameroonian sample (*N* = 90) and one Czech sample (*N* = 80). Results can be found in Table S1.

| Table S1  *Differences in aggressiveness rating and perception accuracy within the two collection sites in Cameroon and the two collection terms in Czech Republic.* | | | | | | | | | | | | | | | | | | | |
| --- | --- | --- | --- | --- | --- | --- | --- | --- | --- | --- | --- | --- | --- | --- | --- | --- | --- | --- | --- |
|  |  | Stimuli |  |  |  |  |  |  |  |  |  |  |  |  |  |  |  |  |  |
|  | Country | African | | | | | |  |  | European | | | |  |  | Mixed | | | |
|  |  | Mean | SD | t | Df | p | Cohen’s d | Mean | SD | t | Df | p | Cohen’s d | Mean | SD | t | Df | p | Cohen’s d |
| Congruence score | Cameroon, Babanki (*N* = 47) | -0.322 | 0.961 | -0.12 | 88 | 0.905 | 0.026 | 0.078 | 0.952 | -0.532 | 88 | 0.596 | 0.112 | 0.249 | 1.052 | 0.655 | 88 | 0.514 | 0.139 |
|  | Cameroon, Buea (*N* = 43) | -0.299 | 0.829 |  |  |  |  | 0.193 | 1.092 |  |  |  |  | 0.106 | 1.011 |  |  |  |  |
|  | Czech Rep., 2015 (*N* = 46) | 0.261 | 0.932 | -1.133 | 78 | 0.261 | 0.256 | -0.208 | 1.006 | 0.812 | 78 | 0.419 | 0.184 | -0.053 | 1.024 | 0.292 | 78 | 0.771 | 0.066 |
|  | Czech Rep., 2017 (*N* = 34) | 0.495 | 0.893 |  |  |  |  | -0.39 | 0.973 |  |  |  |  | -0.117 | 0.914 |  |  |  |  |
| Aggressiveness rating | Cameroon, Babanki (*N* = 47) | -0.519 | 0.952 | -0.862 | 88 | 0.391 | 0.182 | -0.312 | 0.921 | 0.136 | 88 | 0.892 | 0,029 | 0.102 | 0.866 | 0.163 | 88 | 0.871 | 0,035 |
|  | Cameroon, Buea (*N* = 43) | -0.354 | 0.862 |  |  |  |  | -0.283 | 1.087 |  |  |  |  | 0.07 | 0.95 |  |  |  |  |
|  | Czech Rep., 2015 (*N* = 46) | -0.319 | 0.919 | 0.063 | 78 | 0.95 | 0.003 | -0.054 | 1.02 | -0.216 | 78 | 0.829 | 0.049 | 0.375 | 0.953 | 0.766 | 78 | 0.446 | 0.16 |
|  | Czech Rep., 2017 (*N* = 34) | -0.332 | 0. 886 |  |  |  |  | -0.011 | 0.728 |  |  |  |  | 0.221 | 0.776 |  |  |  |  |

**Differences in aggressiveness assessments**

Results regarding perceived aggressiveness, including *post-hoc* tests are summarised in Table S2 and S3. The analysis showed a significant main effect of stimuli category (F _1.693, 753.424_ = 115.379, *p* < 0.001, η_p_^2^ = 0.206) on aggressiveness assessment. All categories of apparent origin significantly differed from each other in perceived aggressiveness, category of apparent Mixed origin was rated the highest in perceived aggressiveness, followed by European origin, and category of African origins was rated as the least aggressive (Table S2).

This main effect was qualified by a statistically significant interaction between stimuli category and rater’s country on the congruence scores (F_5.079, 753.424_ = 7.243, *p* < 0.001, η_p_^2^ = 0.047) (Figure S1).

To understand the nature of the interaction, we ran pairwise comparisons with Bonferroni correction. In the Brazilian sample, perceived aggressiveness were higher for Mixed faces than African and European (*ps* < 0.001), difference between African and European faces was close to formal level of significance (*p* = 0.053); in Cameroon, all categories of faces significantly differed in perceived aggressiveness (*ps* < 0.001) with European faces were rated as most aggressive, followed by Mixed, while African faces received the lowest rating; in the Czech Republic, all categories of faces significantly differed in perceived aggressiveness (*ps* < 0.004) with Mixed faces were rated as most aggressive, followed by European, while African faces received the lowest rating; and in Turkey also, Mixed faces were rated as most aggressive, followed by European, and African faces received again the lowest rating, perceived aggressiveness of all categories of faces significantly differed (*ps* < 0.001) (Table S3).

| Table S2 | | | | | | | |
| --- | --- | --- | --- | --- | --- | --- | --- |
| *Differences in aggressiveness assessments –Stimulus category descriptive statistics and Post Hoc comparison* | | | | | | | |
|  |  | Aggressiveness assessments | |  |  |  |  |
| Stimulus category descriptives | Stimuli | Mean | SE | 95% Confidence intervals | |  |  |
|  |  |  |  | Lower limit | Upper limit |  |  |
|  | African | 3.830 | 0.040 | 3.753 | 3.908 |  |  |
|  | European | 4.108 | 0.039 | 4.032 | 4.184 |  |  |
|  | Mixed | 4.238 | 0.037 | 4.165 | 4.311 |  |  |
|  |  |  |  |  |  |  |  |
|  |  | Bonferroni Post Hoc comparison | | |  |  |  |
| Pairwise comparisons | Stimuli | Mean difference | SE | 95% Confidence intervals | | *p* |  |
|  |  |  |  | Lower limit | Upper limit |  |  |
|  | African - European | -0.277 | 0.032 | -0.353 | -0.201 | < 0.001 |  |
|  | African - Mixed | -0.408 | 0.028 | -0.476 | -0.340 | < 0.001 |  |
|  | European - Mixed | -0.130 | 0.021 | -0.182 | -0.079 | < 0.001 |  |

| Table S3 | | | | | |  |  |
| --- | --- | --- | --- | --- | --- | --- | --- |
| *Differences in aggressiveness assessments – Country x Stimulus category descriptive statistics Interaction summary and Post Hoc comparison* | | | | | | | |
|  |  |  |  |  |  |  |  |
|  | Aggressiveness assessments | | | | | |  |
|  | Country | Stimuli | Mean | SE | 95% Confidence intervals | |  |
|  |  |  |  |  | Lower limit | Upper limit |  |
| Country x stimulus category descriptivesInteraction summary | Brazil | African | 3.821 | 0.070 | 3.684 | 3.958 |  |
|  |  | European | 3.954 | 0.068 | 3.820 | 4.087 |  |
|  |  | Mixed | 4.124 | 0.066 | 3.995 | 4.253 |  |
|  | Cameroon | African | 4.230 | 0.086 | 4.062 | 4.399 |  |
|  |  | European | 4.712 | 0.084 | 4.548 | 4.877 |  |
|  |  | Mixed | 4.562 | 0.081 | 4.403 | 4.720 |  |
|  | Czech Rep. | African | 3.566 | 0.091 | 3.387 | 3.745 |  |
|  |  | European | 3.802 | 0.089 | 3.628 | 3.976 |  |
|  |  | Mixed | 4.082 | 0.086 | 3.914 | 4.250 |  |
|  | Turkey | African | 3.704 | 0.068 | 3.570 | 3.838 |  |
|  |  | European | 3.963 | 0.066 | 3.832 | 4.093 |  |
|  |  | Mixed | 4.185 | 0.064 | 4.059 | 4.311 |  |
|  |  |  |  |  |  |  |  |
|  | Bonferroni Post Hoc comparison | | | | | | |
|  | Country | Stimuli | Mean difference | SE | 95% Confidence intervals | | *p* |
|  |  |  |  |  | Lower limit | Upper limit |  |
| Pairwise comparisons | Brazil | African - European | -0.133 | 0.056 | -0.267 | 0.001 | 0.053 |
|  |  | African - Mixed | -0.303 | 0.050 | -0.422 | -0.183 | < 0.001 |
|  |  | European - Mixed | -0.170 | 0.037 | -0.260 | -0.080 | < 0.001 |
|  | Cameroon | African - European | -0.482 | 0.068 | -0.647 | -0.317 | < 0.001 |
|  |  | African - Mixed | -0.331 | 0.061 | -0.479 | -0.184 | < 0.001 |
|  |  | European - Mixed | 0.151 | 0.046 | 0.040 | 0.261 | 0.004 |
|  | Czech Rep. | African - European | -0.236 | 0.073 | -0.411 | -0.062 | 0.004 |
|  |  | African - Mixed | -0.516 | 0.065 | -0.672 | -0.360 | < 0.001 |
|  |  | European - Mixed | -0.280 | 0.049 | -0.397 | -0.162 | < 0.001 |
|  | Turkey | African - European | -0.259 | 0.054 | -0.389 | -0.128 | < 0.001 |
|  |  | African - Mixed | -0.481 | 0.049 | -0.598 | -0.364 | < 0.001 |
|  |  | European - Mixed | -0.222 | 0.037 | -0.310 | -0.134 | < 0.001 |

Figure S1. Violin and boxplots for differences in perceived aggressiveness.


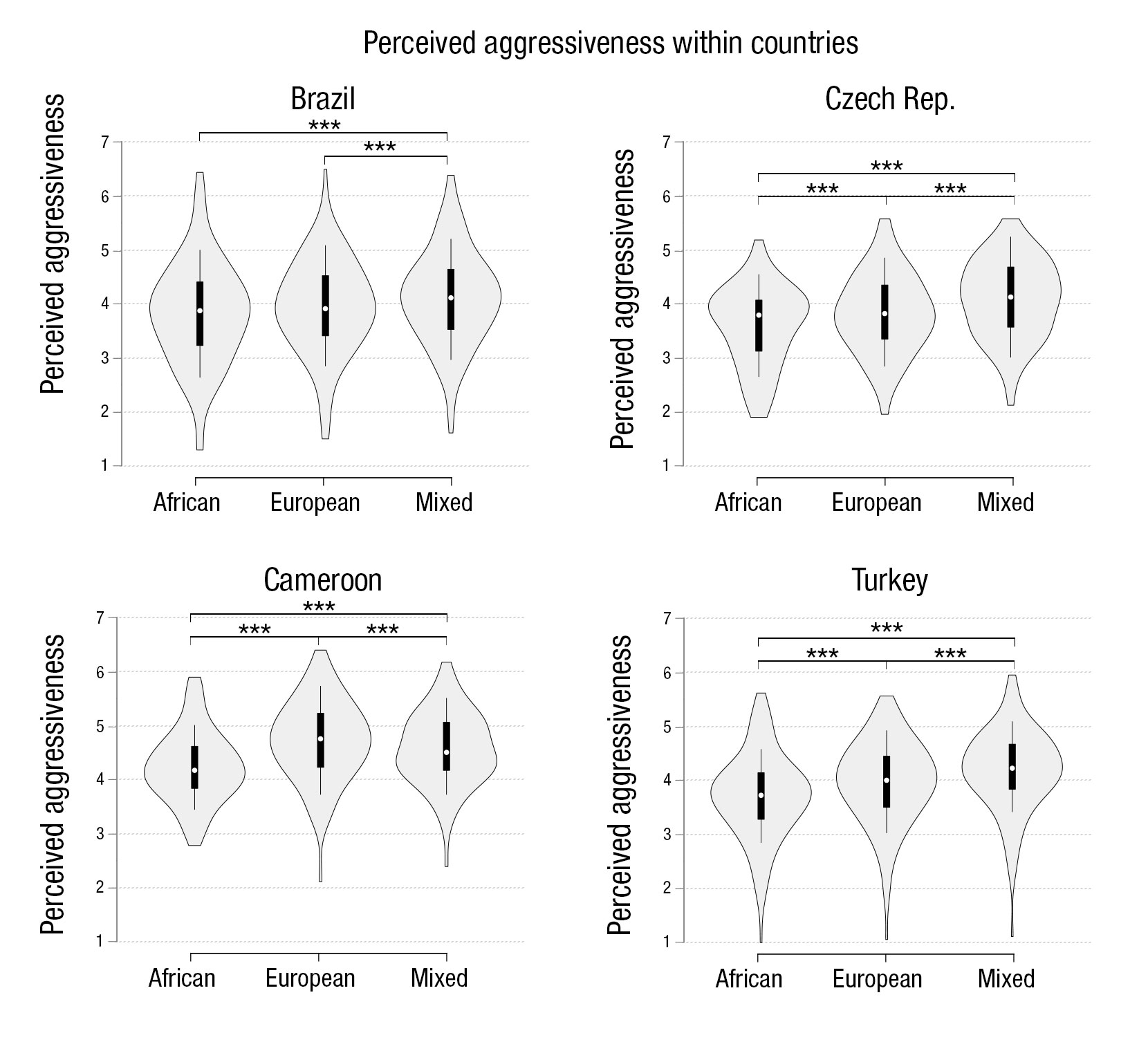


Note: Violin plots represent perceived aggressiveness. Box limits indicate the 2^5th^ and 75^th^ percentiles, whiskers extend 1.5 times the interquartile range from the 25^th^ and 75^th^ percentiles. Asterisks stands for significance levels; *** for p < 0.001, ** for p < 0.01, * for p < 0.05.

| Table S4 | | | | | | | | | | | | | | |
| --- | --- | --- | --- | --- | --- | --- | --- | --- | --- | --- | --- | --- | --- | --- |
| *Results for perceived aggressiveness and congruence scores in Czech Sample 2013* | | | | | | | | | | | | | | |
|  |  |  |  |  | Two tailed One-Way ANOVA | | | | Bonferroni Post-hoc comparisons | | | | | |
| Country | Scale | Stimuli |  |  |  |  |  |  | African × European | | African × Mixed | | European × Mixed | |
|  |  |  | Mean | SD | F | Df | p | Partial η2 | p | Cohen's d | p | Cohen's d | p | Cohen's d |
| Czech Republic 2013  (N = 100) | Perceived Aggressiveness | African | 0.028 | 0.996 | 3.204 | 1, 297 | 0.042 | 0.021 | 1 | 0.130 | 0.36 | 0.227 | 0.038 | 0.354 |
|  |  | European | 0.161 | 1.052 |  |  |  |  |  |  |  |  |  |  |
|  |  | Mixed | -0.19 | 0.925 |  |  |  |  |  |  |  |  |  |  |
|  | Congruence score | African | -0.579 | 0.904 | 43.834 | 1, 297 | < 0.001 | 0.049 | <0.001 | 0.664 | <0.001 | 1.275 | <0.001 | 0.686 |
|  |  | European | -0.009 | 0.811 |  |  |  |  |  |  |  |  |  |  |
|  |  | Mixed | 0.588 | 0.926 |  |  |  |  |  |  |  |  |  |  |

The Dataset for Czech 2013 sample is available on the Open Science Framework, https://osf.io/wec83/?view_only=8d160ad438ab4d89868681bf512f5a31.
